# Close Vicinity of PrP Expressing Cells (FDC) with Noradrenergic Fibers in Healthy Sheep Spleen

**DOI:** 10.1155/2001/40871

**Published:** 2001

**Authors:** A. Bencsik, S. Lezmi, G. Hunsmann, T. Baron

**Affiliations:** 1 AFSSA Unité de Virologie-ATNC 31 avenue Tony Garnier BP 7033 Lyon cedex 07 69342 France; 2 Deutsches Primatenzentrum Dept Virology and Immunology Göttingen D-37077 Germany

**Keywords:** FDC, Lymphoid tissue, Neuroinvasion, Noradrenergic fibers, Prion, Sheep

## Abstract

In naturally and experimentally occurring scrapie in sheep, prions invade the immune system and replicate in lymphoid organs. Here we analysed immunohistochemically, in seven spleens of 6-month-old healthy sheep, the nature of the cells expressing prion protein (PrP) potentially supporting prion replication, as well as their relationship with autonomic innervation. PrP was identified using either RB1 rabbit antiserum or 4F2 monoclonal antibody directed against AA 108–123 portion of the bovine and AA 79–92 of human prion protein respectively. Using double labelling analysis, we demonstrated that PrPc is expressed by follicular dendritic cells using a specific monoclonal antibody (CNA42). We also showed the close vicinity of these PrP expressing cells with noradrenergic fibers, using a polyclonal tyrosine hydroxylase antibody. Our results may help the study of the cellular requirements for the possible neuroinvasion from the spleen.


*Developmental Immunology*, 2001, Vol. 8(3-4), pp. 235-241
Reprints available directly from the publisher
Photocopying permitted by license only

© 2001 OPA (Overseas Publishers Association)
N.V. Published by license under
the Harwood Academic Publishers imprint,
part of The Gordon and Breach Publishing Group,
a member of the Taylor and Francis Group

### Close Vicinity of PrP Expressing Cells (FDC) with Noradrenergic Fibers in Healthy Sheep Spleen

A. BENCSIK[a*], S. LEZMI[a], G. HUNSMANN[b] and T. BARON[a]

[a]*AFSSA, Unité de Virologie-ATNC, 31 avenue Tony Garnier, BP 7033, 69342 Lyon cedex 07, France and* [b]*Deutsches Primatenzentrum, Dept Virology and Immunology, D-37077 Göttingen, Germany*

In naturally and experimentally occurring scrapie in sheep, prions invade the immune system and replicate in lymphoid organs. Here we analysed immunohistochemically, in seven spleens of 6-month-old healthy sheep, the nature of the cells expressing prion protein (PrP) potentially supporting prion replication, as well as their relationship with autonomic innervation. PrP was identified using either RB1 rabbit antiserum or 4F2 monoclonal antibody directed against AA 108–123 portion of the bovine and AA 79–92 of human prion protein respectively. Using double labelling analysis, we demonstrated that PrPc is expressed by follicular dendritic cells using a specific monoclonal antibody (CNA42). We also showed the close vicinity of these PrP expressing cells with noradrenergic fibers, using a polyclonal tyrosine hydroxylase antibody. Our results may help the study of the cellular requirements for the possible neuroinvasion from the spleen.

*Keywords:* FDC, Lymphoid tissue, Neuroinvasion, Noradrenergic fibers, Prion, Sheep

### INTRODUCTION

The transmissible spongiform encephalopathies (TSEs) are neurodegenerative disorders in which the immune system plays a critical role in the pathogenesis, not only as the initial peripheral site of replication of the TSE's agent, but also with regard to its implication in the neuroinvasion process (Aguzzi, 1997; Beekes et al., 1996; Race et al., 1998). Both nervous and immune systems have one of the identified cellular requirements for the replication of the TSE's agent that is cellular PrP expressing cells (Brandner et al., 1996; Büeler et al., 1993; Horiuchi et al., 1995; McBride et al., 1992). Within lymphatic tissues the germinal centre microenvironment has been more particularly incriminated in peripheral prion replication with the implication of B lymphocytes / follicular dendritic cells (FDCs) (Kitamoto et al., 1991; Klein et al., 1997; Raeber et al., 1999a; Raeber et al., 1999b). How TSE's agent propagates from lymphatic tissues to Central Nervous System (CNS) is unclear but autonomic nerve system that innervates the lymphoid organs may contribute to their dissemination from the spleen to the CNS (Fried et al., 1986). The comparative analysis of the PrP expressing cells topography with the noradrenergic fibers of autonomic nerve system within the spleen may help to understand how the infectivity spreads in the course of natural infection. The purpose of our work was thus to determine in sheep spleen the immunohistochemical repartition of noradrenergic fibers and PrP expressing cells.

* Corresponding author: Phone number: (33) 04 78 72 65 43 Fax: (33) 04 78 61 91 45 E-mail: a.bencsik@lyon.afssa.fr

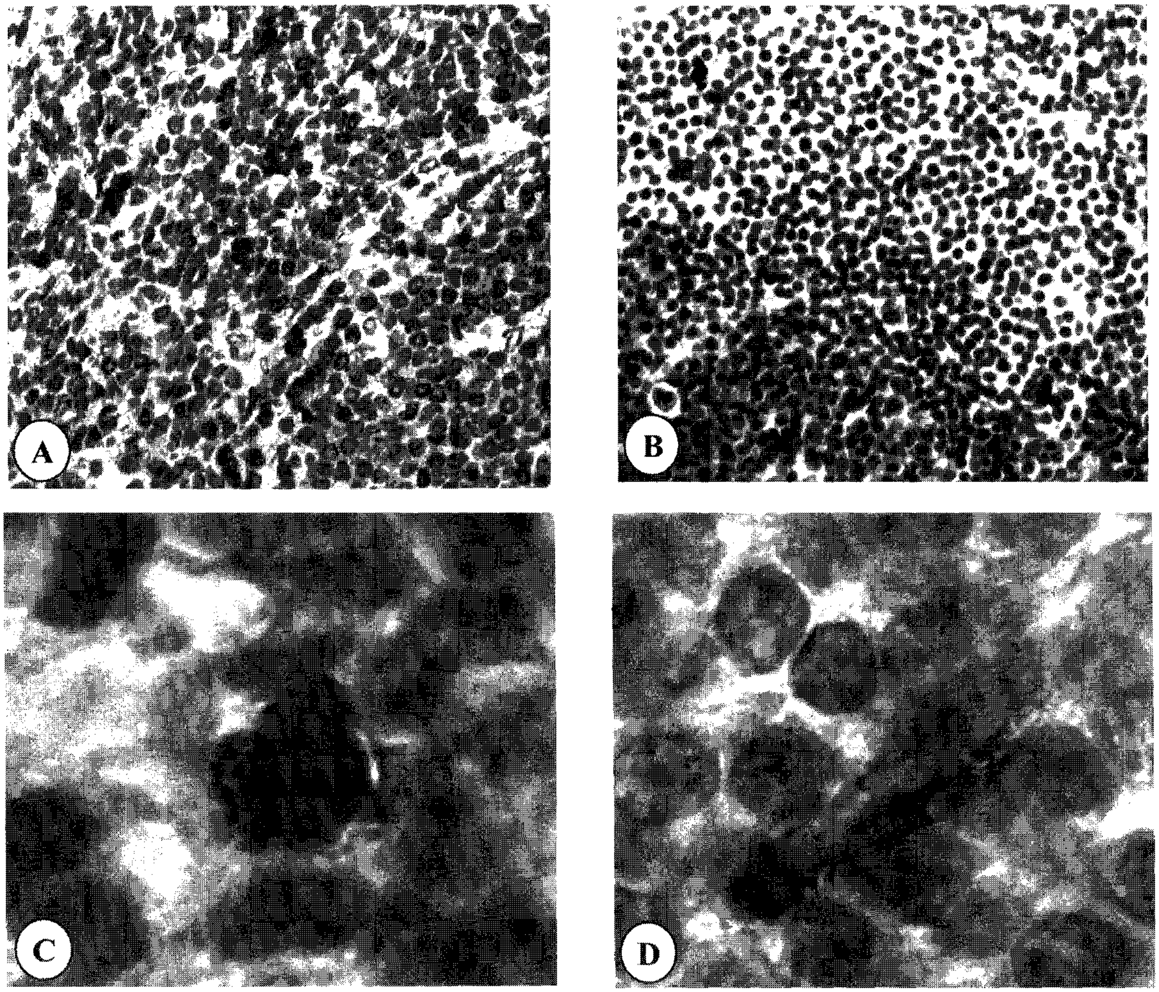

FIGURE 1 PrP expressing cells detected using 4F2 (A, C, D) within the spleen of healthy lambs. Note the density of PrP expressing cells in the coronal area where FDCs are also seen using CNA42 (B). In the limit of the mantle zone, PrP expressing cells are not labelled with CNA42 and present a more fusiform shape (D)

### RESULTS

### PrP Expressing Cells in the Spleen of Lambs

PrP was detectable using 3 different antibodies directed against two different regions of PrP (4F2, RS1, RB1), within the primary and secondary lymphoid follicles of the spleen (Fig 1A). The number of 4F2 labelled follicles varied between the lambs from 16% to 100% depending mostly on the fixatives. PrP staining consisted more frequently as a fine deposit redrawing the cell membrane (Fig 1 C&D). PrP was never seen in cortical areas, in red pulp, nor in blood vessels. In order to validate the cellular nature of PrP, we controlled that this PrP immunostaining disappeared when a combination of different pre-treatments was previously applied. The specificity of PrP immunostaining was also checked using an irrelevant antibody (GFAP) which didn't give such a staining. Sections in which the primary antibody was omitted were also used to evaluate the background staining, which was inexistent.

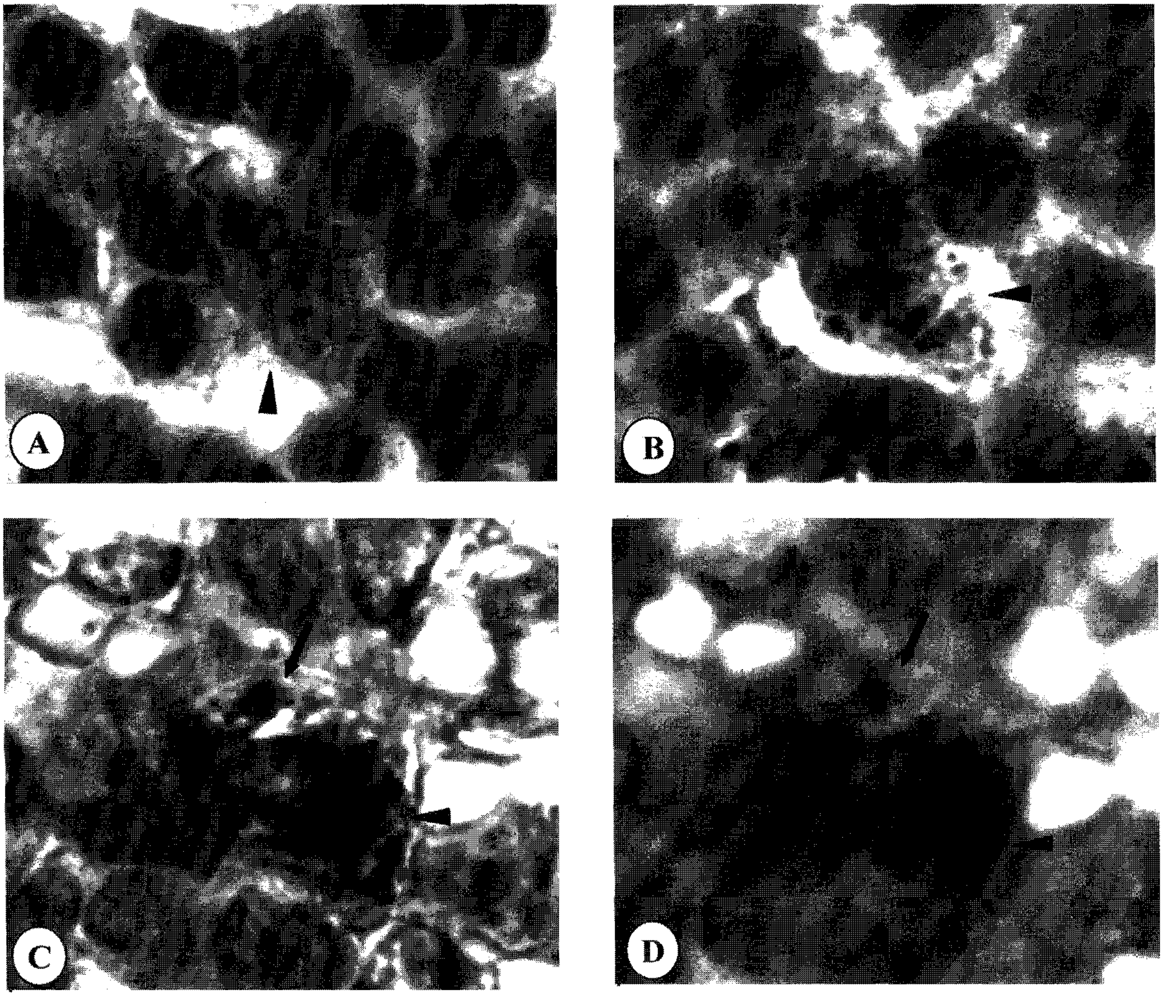

FIGURE 2 FDCs identified by MGG staining (head arrow)(A) or by CNA42 monoclonal antibody (B), are associated with lymphocytes in lymphoid follicles. Two different focus of the same cell within a lymphoid follicle, identified as an FDC (head arrow, brown deposit) using CNA42, and PrP expressing cell using RB1 (arrow, red deposit) both markers located in the cytoplasm of the same cell (C, D)

**FDCs Express PrP in the Spleen**

The Follicular Dendritic Cells (FDCs) were identifiable using May-Grünwald-Giemsa (MGG) staining (Fig 2A) and with CNA42 antibody (Fig 1B). In both primary and secondary lymphoid follicles FDCs stained with MGG have a big nucleus, with a pink cytoplasm, a dendritic morphology and with an approximate size of 50 to 100 μm of diameter. The CNA42 immunolabelling pattern was cytoplasmic, more intense along the cell membrane, redrawing many cell processes associated with neighbouring lymphocytes (Fig 2B). No FDC labelling was seen in the red pulp or in the blood vessels area. PrP detected using RB1 was seen within FDCs in our double labelling experiments (Fig 2C&D).

**Autonomic Innervation of the Spleen**

Noradrenergic innervation was recognisable in the spleen using TH antibody. Noradrenergic fibers were

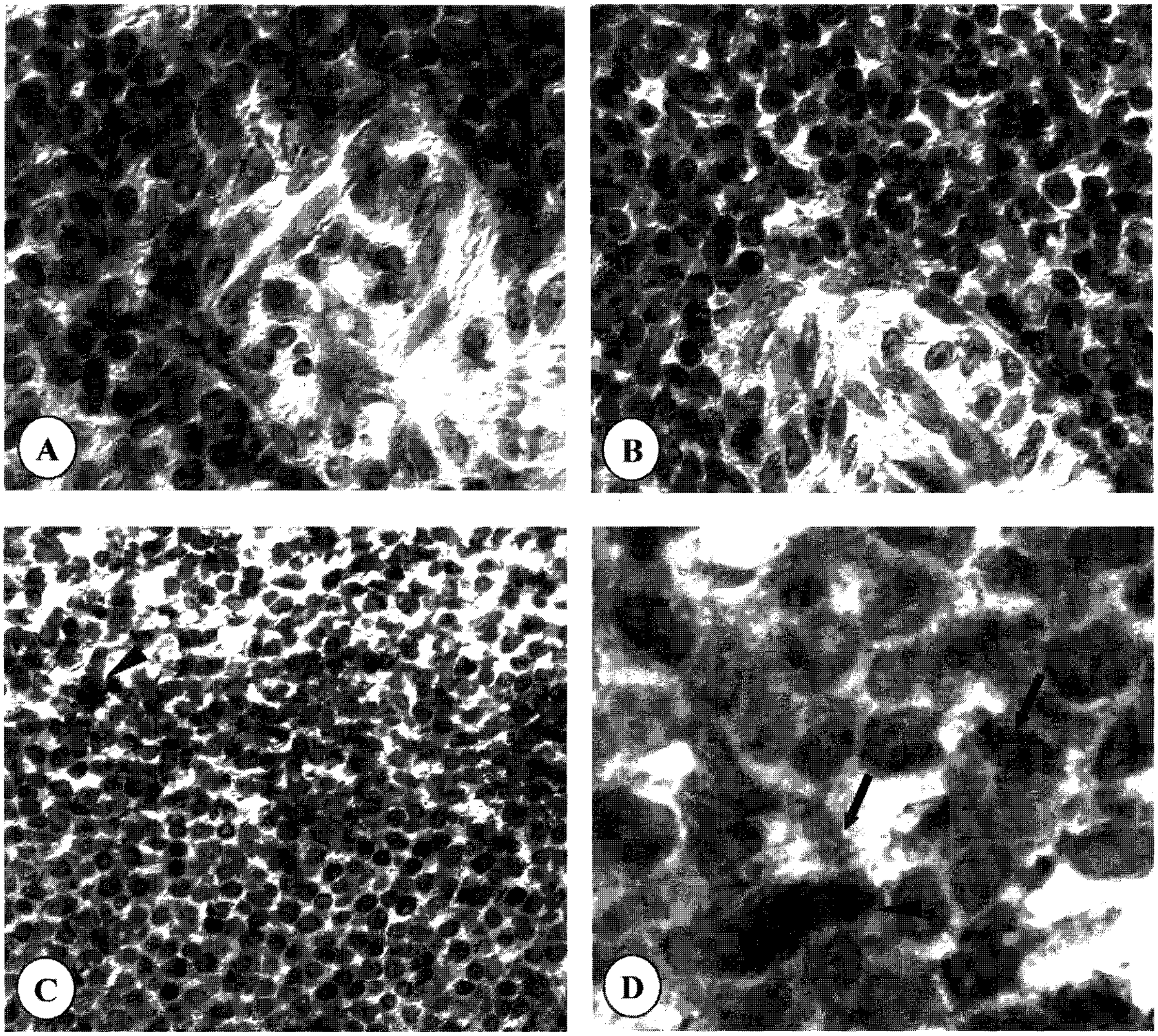

FIGURE 3 Sympathetic innervation of the spleen. Noradrenergic fibers identified using anti-TH antibody (red deposit) around blood vessels (A) and near several immune cells (B). In double labelling experiments, noradrenergic fibers (red deposit, arrow) were detected at proximity of PrP expressing cells (brown deposit, head arrow) revealed using 4F2 (C&D)

located within the trabeculae, the vasculature (Fig 3A) and the capsule of the spleen. Within the white pulp, noradrenergic fibers were seen in the marginal zone and the mantle zone near immune cells (Fig 3B). The immunostaining consisted of fine homogenous cytoplasmic deposits redrawing some processes. Using double labelling experiments, noradrenergic fibers were seen in the white pulp of the spleen at proximity of PrP expressing cells detected with 4F2 (Fig 3C&D).

### DISCUSSION

In transmissible spongiform encephalopathies, prion replication occurs in the lymphoid system of sheep with scrapie as well as in rodent models of scrapie (O'Rourke et al., 1998; Race and Ernst, 1992; Raeber et al., 1999a). The precise cell types involved in this lymphoreticular prion replication and transport to the Central Nervous System (CNS) remains unclear although several lines of evidence argue for a key role for Follicular Dendritic Cells (FDCs) as well as an involvement of B lymphocytes (Klein et al., 1997; McBride et al., 1992). It is important to identify the type of cells involved not only in propagating but also carrying prion infectivity or both in peripheral tissues. Since it has been shown that prion replication needs the expression of cellular form of PrP expression (Büeler et al., 1993), we have investigated the nature of PrP expressing cells within the spleen of healthy lambs. We demonstrated that the cellular form of PrP is expressed by FDCs not only on similarities of morphology and localisation of PrPc expressing cells but mostly because we found that FDCs and PrP markers were co-localised in the same cell in our double labelling experiments. It was noticeable that every PrP expressing cells are not all typically FDCs particularly because they didn't share the same location (Imai and Yamakawa, 1996). This may reflect the fact that there may be several types of FDCs and only a part of them are identifiable using CNA42 (Schuurman et al., 1993). This may also indicate that these cells could be B lymphocytes since they have been described as another major type of immune cell involved in peripheral prion replication. In our case, B lymphocytes couldn't be identified yet, particularly by a specific antibody to mammalian B-lymphocytes compared to mouse or cat spleen where B lymphocytes were easily detectable (data not shown). But B lymphocytes could be implicated in the TSE's pathogenesis without expressing PrP by themselves as well (Klein et al., 1998). Interestingly, our study brings evidences of the existence of at least another type of cells beside typical FDCs that are not CNA42 labelled. These cells, identifiable only the basis of their more fusiform shape and specific location at the periphery of the mantle zone of the follicles strongly reminds the description of the Fibroblastic Reticulum Cells (FRC) (Imai and Yamakawa, 1996). This is not necessarily in contradiction with the FDC nature since it is possible that under some factors, these unmature cells may be able to differentiate into FDCs. Beside the FDCs we also showed the possible involvement of autonomic nerve system. Actually, the presence of autonomic nerve fibers in lymphoid tissues provides an anatomical link between the nervous and immune systems. This may represent a part of the particular channel of communication implicated in prion spread in the body. The present immunohistochemical study provides direct evidence that noradrenergic fibers, revealed by the presence of one of the monoaminergic synthesising enzymes, the tyrosine hydroxylase as marker (Björklund and Hökfelt, 1985), are present within the ovine spleen. The great majority of the noradrenergic fibers were detected at proximity of the splenic nerve, around blood vessels, which is consistent with a classical role of blood pressure control or regulation of spleen contraction via the noradrenergic innervation detected in the capsule (Fried et al., 1986). More interestingly, the colocalisation of noradrenergic fibers with PrP expressing cells within the follicular area raises the question of another type of function of sympathetic nerves in the spleen, like an immunomodulating function. Thus this close vicinity of noradrenergic fibers with PrP expressing cells will have to be analysed in sheep affected with scrapie. This analysis is under progress and will allow evaluating the potential involvement of noradrenergic innervation in the transport of TSE's agents from the spleen to the CNS.

### MATERIALS AND METHODS

### Tissues

The spleens of seven healthy lambs (6 month-old) were immersed in different buffered fixatives (10% formalin (n=1), 4% (n=3) or 2% (n=3) paraformaldehyde). Once fixed, spleen samples were rinsed for a week in cold phosphate buffer (PB 0.1 M, pH 7.4) then routinely dehydrated and embedded in paraffin. To ensure adhesion, sections (3 or 5 μm) of spleen were collected onto pre-treated glass slides (Polylisin or StarFrost, Fischer Scientific, Pittsburgh, PA) and baked overnight at 57°C.

TABLE I Primary antibodies used, to detect PrP, FDCs, NA fibers, and glial cells

| *Antibodies* | *Species* | *Dilutions* | *Origin* |
|---|---|---|---|
| RS1-RB1 (PrPov 98–113/PrPbov 108–123) | Rabbit | 1/100 | AFSSA, Lyon, France |
| 4F2 (PrPhu 79–92) | Mouse | 1/200 | German Primate Center, Göttingen, Germany |
| CNA42 (Follicular Dendritic Cells, FDC) | Mouse | undiluted | Hôpital Toulouse, France |
| TH (Tyrosine hydroxylase, NA cells) | Rabbit | 1/200 | Jacques Boy Institute |
| GFAP (Glial cells) | Rabbit | 1/200 | Dako |

### Antibodies

To detect the PrP in the lambs, we used the 4F2 monoclonal antibody (kindly provided by J. Grassi, CEA/SPI, Saclay, France) and two rabbit antisera RS1 and RB1 (Table I). These antisera are specific for PrP as demonstrated previously by Western blot applied on purified prion protein from scrapie affected sheep brain (Madec et al., 1998). The FDCs were identified using a monoclonal antibody, the CNA42 (courtesy of Pr Delsol, Hôpital Toulouse, France) (Delsol et al., 1993). Noradrenergic fibers were identified using a rabbit polyclonal antibody against the tyrosine hydroxylase (TH) (Institut Jacques Boy, France), the rate limitating enzyme in the synthesis of noradrenaline. We also used as control of PrP specificity a rabbit anti-GFAP polyclonal antibody (Dako).

### Staining Procedure

The slides were dewaxed and rehydrated in water, then used for immunohistochemical analysis. Every step was done at room temperature. Endogenous peroxidase activity was blocked with 10 min incubation in 3% hydrogen peroxide in PBS 0.1M. Unspecific antigenic sites were blocked by a 30 min step in blocking reagent (Roche-Boehringer). Then the sections were incubated overnight with the primary antibody (GFAP or TH, Table I). The spleen sections were rinsed before the detection of the primary antibody using the ABC system (Vector), succinctly first using the biotinylated anti-rabbit antibody (30 min) and secondly with a peroxydase-labelled avidin-biotin complex (ABC) (30 min). These steps were followed by washes and finally peroxydase was revealed by immersing the sections in aminoethylcarbazole solution (AEC, Dako) giving a red deposit or in DAB intensified with NiC12 (Biosys) giving a dark brownish deposit. For PrP or FDC immunodetection slides were first treated in PBS (0.1 M pH 7.4) for 3 min at 450W in a microwave oven in order to enhance the staining by unmasking epitopes of FDCs and PrP. Then the tyramide signal amplification method was applied as specified by the manufacturers (kit TSA, NEN Dupont, Le Blanc Mesnil, France). Briefly, unspecific antigenic sites were blocked using the TNB (protein blocking reagent). After eliminating the excess of TNB, slides were placed for 24 to 48 h with the primary antibody diluted in TNB (Table I). After a wash with TNT solution, the secondary antibody (anti-mouse IgG biotinylated, Science technology or anti-rabbit IgG biotinylated, ABC Vector) diluted at 1/100 in TNB was applied for 1h. Without any wash the following step was 30 min of incubation with SA-HRP (1/100 in TNB). Once rinsed, slides were subjected to the enhancement step, 10 min with biotinyl tyramide (1/50 in amplification diluant). A second 30 min of incubation in SA-HRP preceded the final step of immunostaining. The presence of antigens being revealed either with AEC or DAB. To assess the specificity of PrP immunostaining several pre-treatments such as 30 min hydrated autoclaving (immersed in PBS 0.1M pH 7.4, for 3 successive cycles of 11 min at 121°C in a pressure cooker), immersion for 15 min in 98% formic acid (Merck), proteinase K treatment (Roche-Boehringer, 20 μg/ml in PBS 0.1M, pH7.4, 37°C, for 15 min), or guanidium thiocyanate (Sigma, 4M, for 60 min, at room temperature) leads to the destruction of cellular form of PrP.

### Double Staining Procedure

To identify the nature of PrP expressing cells, we have developed a double immunostaining procedure using both RB1 antiserum and CNA42 monoclonal antibody. We also applied the double immunostaining procedure to visualise noradrenergic fibers (anti-TH antibodies) and PrP expressing cells (4F2). Following the first immunohistochemistry procedure, the second one was performed, beginning with a new blocking step (TNB, 30 min). Then the procedure applied was the same as described before. To reveal the histological organisation of the spleen, a very weak staining was obtained with aqueous haematoxylin. Finally, slides were mounted with Crystal Mount medium and observed under a microscope coupled to an image analysis workstation (βiocom, Les Ulis France).

### *Acknowledgements*

This work was supported in parts by grants from "Programme de Recherches sur les ESST et les Prions". Stéphane LEZMI was financially supported by a grant from the "Académie Nationale de Médecine".

We thank T. Marchal (ENVL) for helpful discussion concerning FDCs

### *References*

Aguzzi A. (1997). Neuro-immune connection in spread of prions in the body? Lancet **349**:742–743.

Beekes M., Baldauf E., and Dringer H. (1996). Sequential appearance and accumulation of pathognomonic markers in the central nervous system of hamsters orally infected with scrapie. J. Gen. Virol. **77**:1925–1934.

Björklund A., and Hökfelt T. (1985). "Handbook of chemical neuroanatomy – Classical transmitters in the CNS." Elsevier, Amsterdam.

Brandner S., Isenmann S., Raeber A., Fischer M., Sailer A., Kobayashi Y., Marino S., Weissmann C., and Aguzzi A. (1996). Normal host prion protein necessary for scrapie-induced neurotoxicity. Nature **379**:339–343.

Büeler H., Aguzzi A., Sailer A., Greiner R.A., Autenried P., Aguet M., and Weissmann C. (1993). Mice Devoid of PrP Are Resistant to Scrapie. Cell **73**:1339–1347.

Delsol G., Meggetto F., Brousset P., Cohen-Knafo E., Al Saati T., Rochaix P., and al, (1993). Relation of follicular dendritic reticulum cells to Reed-Sterberg cells of Hodgkin's disease with emphasis on the expression of CD21 antigen. American Journal of Pathology **142**:1729–1738.

Fried G., Terenius L., Brodin E., Efendic S., Dockray J., Fahrenkrug J., Goldstein M., and Hökfelt T. (1986). Neuropeptide Y, enkephalin and noradrenaline coexist in sympathetic neurons innervating the bovine spleen. Biochemical and immunohistochemical evidence. Cell and Tissue Research **243**:495–508.

Horiuchi M., Yamazaki N., Ikeda T., Ishiguro N., and Shinagawa M. (1995). A cellular form of prion protein (PrPC) exists in many non-neuronal tissues of sheep. J. Gen. Virol. **76**:2583–2587.

Imai Y., and Yamakawa M. (1996). Morphology, function, and pathology of follicular dendritic cells. Pathol. Int **46**:807–833.

Kitamoto T., Muramoto T., Mohri S., Doh-Ura K., and Tateishi J. (1991). Abnormal Isoform of Prion Protein Accumulates in Follicular Dendritic Cells in Mice with Creutzfeldt-Jakob Disease. Journal of Virology **65**:6292–6295.

Klein M. A., Frigg R., Flechsig E., Raeber A. J., Kalinke U., Bluethmann H., Bootz F., Suter M., Zinkernagel R. M., and Aguzzi A. (1997). A crucial role for B cells in neuroinvasive scrapie. Nature **390**:687–690.

Klein M. A., Frigg R., Raeber A. J., Flechsig E., Hegyi I., Zinkernagel R. M., Weissmann C., and Aguzzi A. (1998). PrP expression in B lymphocytes is not required for prion neuroinvasion. Nature Medicine **4**:1429–1433.

Madec J.-Y., Groschup M. H., Buschmann A., Belli P., Calavas D., and Baron T. (1998). Sensitivity of the Western blot detection of prion protein PrPres in natural sheep scrapie. Journal of Virological Methods **75**:169–177.

McBride P. A., Ekelenboom P., Kraal G., Fraser H., and Bruce M. E. (1992). PrP protein is associated with follicular dendritic cells of spleens and lymph nodes in uninfected and scrapie-infected mice. Journal of Pathology **168**:413–418.

O'Rourke K. I., Bazsler T. V., Parish S. M., and Knowles D. P. (1998). Preclinical detection of PrPsc in nictitating membrane lymphoid tissue of sheep. The Veterinary Record **142**:489–491.

Race R., Jenny A., and Sutton D. (1998). Scrapie infectivity and proteinase K-resistant prion protein in sheep placenta, brain, spleen, and lymph node: Implications for transmission and antemortem diagnosis. Journal of Infectious Diseases **178**:949–953.

Race R. E., and Ernst D. (1992). Detection of proteinase K-resistant prion protein and infectivity in mouse spleen by 2 weeks after scrapie agent inoculation. Journal of Virology **73**:3319–3323.

Raeber A. J., Klein M. A., Frigg R., Flechsig E., Aguzzi A., and Weissmann C. (1999a). PrP-dependent association of prions with splenic but not circulating lymphocytes of scrapie-infected mice. The EMBO Journal **18**:2702–2706.

Raeber A. J., Sailer A., Hegyi I., Klein M. A., Rulicke T., Fischer M., Brandner S., Aguzzi A., and Weissmann C. (1999b). Ectopic expression of prion protein (PrP) in T lymphocytes or hepatocytes of PrP knockout mice is insufficient to sustain prion replication. Proceedings of the National Academy of Sciences of the United States of America **96**:3987–3992.

Schuurman H. P., Joling P., van Wichen D. F., Rademarkers L. H. P. M., Parmentier H. K., de Weger R. A., and Goudsmit J. (1993). "Follicular dendritic cells in lymph nodes from patients after HIV-1 infection. A crucial target cell type in the infection process." In Dendritic cells. Vol 3. Imai Y., Asai J., Iijima M., Shamoto M., Tamafi K. and Fuse Y. (eds). Sakabe Publishing House Inc. Yamagata, pp. 1–27.

